# Using institutional ethnography to analyse animal sheltering and protection I: Animal protection work

**DOI:** 10.1017/awf.2023.39

**Published:** 2023-06-09

**Authors:** Katherine E Koralesky, Janet M Rankin, David Fraser

**Affiliations:** 1 Animal Welfare Program, 2357 Main Mall, University of British Columbia, Vancouver, BC, Canada V6T1Z4; 2 Faculty of Nursing, University of Calgary, 2500 University Drive NW Calgary, AB, Canada T2N1N4

**Keywords:** Animal law, animal welfare, intervention, legal, policy, regulation

## Abstract

Animal protection laws exist at federal, provincial and municipal levels in Canada, with enforcement agencies relying largely upon citizens to report concerns. Existing research about animal protection law focuses on general approaches to enforcement and how legal terms function in the courts, but the actual work processes of animal law enforcement have received little study. We used institutional ethnography to explore the everyday work of Call Centre operators and Animal Protection Officers, and we map how this work is organised by laws and institutional polices. When receiving and responding to calls staff try to identify evidence of animal ‘distress’ as legally defined, because various interventions (writing orders, seizing animals) then become possible. However, many cases, such as animals living in deprived or isolated situations, fall short of constituting ‘distress’ and the legally mandated interventions cannot be used. Officers are also constrained by privacy and property law and by the need to record attempts to secure compliance in order to justify further action including obtaining search warrants. As a result, beneficial intervention can be delayed or prevented. Officers sometimes work strategically to advocate for animals when the available legal tools cannot resolve problems. Recommendations arising from this research include expanding the legal definition of ‘distress’ to better fit animals’ needs, developing ways for officers to intervene in a broader range of situations, and more ethnographic research on enforcement work in jurisdictions with different legal systems to better understand how animal protection work is organised and constrained by laws and policies.

## Introduction

Animal protection law aims to protect animals from harmful human actions. In Canada, the federal Criminal Code prohibits wilful (including reckless) acts that cause unnecessary pain, suffering or injury to animals, and includes a ban on animal fighting or baiting. Most provinces have laws that prohibit people from causing or permitting an animal to be in ‘distress’ although definitions of this term vary and have broadened over time (Fraser *et al.* 2018). Approaches to law enforcement also vary across jurisdictions (Fraser *et al.* 2018) with Societies for the Prevention of Cruelty to Animals (SPCAs), government agencies, municipal agencies and police taking the lead, depending on the location. Municipal laws, which differ across jurisdictions, broadly regulate matters relating to animals in the public interest including dog licencing and bite prevention, but also may contain provisions to protect animal welfare. Enforcing these various laws relies substantially upon members of the public, as well as veterinarians and others who frequently interact with animals, to report concerns.

Research on the enforcement of animal protection laws has tended to focus on three broad topics. First, researchers have analysed how legal terms structure what happens when laws are applied. For example, Gacek (2019) analysed summaries of court cases focused on the term ‘wilful neglect’ in the Criminal Code, and Ziegler (2019) examined the powers of the provincial Manitoba Animal Care Act compared to the Canadian Charter of Rights and Freedoms. Verbora (2015) analysed parliamentary proceedings regarding changes to the animal section of the Criminal Code, some of which centred on disagreements regarding the definition of ‘animal’ and the implications of animals being termed a form of property. Fraser *et al.* (2018) provided an overview of federal and provincial laws in Canada focusing on how laws define terms such as ‘distress’ and ‘duty of care’ (for an updated overview, see Duval 2021). Thus, although topics vary, these researchers analyse how legal terms function and influence the actions that law enforcement organisations can take for animals.

Second, research has examined some organisational approaches to enforcement. Coulter and Campbell (2020) conducted interviews and document analysis to depict how government agencies enforce animal protection law in Manitoba. Coulter and Campbell (2020), plus a comment by Lees (2018) and articles by Whiting *et al.* (2006) and Whiting (2009), concluded that government-led enforcement, as used in Manitoba, is preferable to enforcement by charitable organisations as it avoids potential conflict of interest. In Australia, Morton and Whittaker (2022) reviewed state and territorial legislation and Morton *et al.* (2020) applied a framework called the ‘enforcement gap’ and identified that various factors, for example, inconsistent legal definitions and reliance on charitable organisations to enforce the law, contributed to discrepancies between the law and actual enforcement practices.

Third, some articles theorise about the relationship between individuals and organisations involved with animal protection law enforcement. Daniell (2002) stressed the importance of a partnership between veterinarians and the SPCA, and Whiting *et al.* (2006) and Whiting (2009) suggested that veterinarians lead on legislation and enforcement activities. The Canadian Veterinary Medical Association and American Veterinary Medical Association guidelines on how to identify and report abuse (Arkow *et al.* 2011; CVMA 2018) are featured in articles geared toward veterinarians (Arkow 2015; Marion 2015). Alleyne *et al.* (2019) used the theory of perceived self-efficacy (a theory grounded in an individual’s belief in themselves and their capabilities) to understand how veterinarians make decisions about reporting abuse based on factors including specialised training and previous experiences with suspecting and reporting abuse. Finally, some articles have discussed institutional collaboration on animal protection law. This includes social network analyses (Reese & Ye 2017), examining institutional thinking and organisational discourse (Stoddart *et al.* 2016) and discussing historical connections between human and animal social work services (Zilney & Zilney 2005; Hoy-Gerlach *et al.* 2019).

This paper uses a different approach to understand how animal protection law organises what can and cannot be done for animals through law enforcement. It explores the actual, everyday work activities of Provincial Call Centre operators (henceforth called operators) and Animal Protection Officers and Special Provincial Constables (henceforth called officers) when they receive calls of concern. This paper presents the case of a dog named Henry and six other dogs he lived with. Henry and the dogs were the subject of investigations over several years before they were eventually seized and brought to a shelter. This case thus provides an entry-point into the everyday work of operators and officers. With a focus on operator and officer work, we used institutional ethnography to discover a series of tensions that arise over animals living in situations that are of concern but where enforcement activities are organised and constrained by municipal, provincial and federal laws, especially the provincial Prevention of Cruelty to Animals (PCA) Act which directs operators and officers to look for and identify animal ‘distress’ as it is defined in the law (see ‘Definitions’ in British Columbia Government 2023). Our use of ethnographic data, and our focus on tensions that arise, provided a different way of viewing the everyday work of officers and operators and the different types of knowledge they have about how to accomplish their work.

## Materials and methods

### Ethical approval

The University of British Columbia Behavioural Research Ethics Board (#H19-00009) and the British Columbia SPCA (BC SPCA) approved all research procedures.

### Institutional ethnography

Institutional ethnography (IE) is an approach to inquiry that aims to discover and explicate how routine, everyday work practices carried out by people are organised by institutional processes (Smith 1987, 1990, 2005). IE can be applied to diverse institutional settings and its ontology is grounded in examining material, observable processes people do and how that work co-ordinates with other people’s work. (Smith 2005; p 52, 227). Regarding work, institutional ethnographers focus on what Smith refers to as ‘work knowledge’, which is local, experiential knowledge that people have about their work, including how they do the work, how they know what they need to do, and how they feel about it (Smith 2005; p 151, 155). IE directs attention to discovering instances when institutional goals (such as those embedded in animal protection law) and what happens in people’s everyday work (in this case the work of operators and officers) do not quite match up. The discovery of such tensions determines the direction of inquiry (Smith 2005; p 38). With this focus on discovering tensions in everyday work, the research questions addressed in IE are often broadly topical, focused on ‘happenings’ rather than grounded in theory.

Animal protection involves many individuals – frontline shelter staff, operators, officers, administrators, directors, police, social service workers and animals. These individuals occupy different ‘standpoints’, or locations in the institution and thus have different understandings of the institution and how it works (Smith 1987; p 107). In IE, researchers place themselves at a specific standpoint, and this standpoint location is the ‘point of entry’ into inquiry (Smith 1990; p 5, Smith 2005 p 10). Although IE projects typically take the standpoint of people, we began inquiry from the standpoint of animals who have become involved with animal protection.

Taking the standpoint of animals involved noting what they were doing when we observed them during ‘ride-alongs’ (i.e. accompanying officers as a passenger in the vehicle as they worked). We recorded fieldnotes about how animals communicated behaviourally (e.g. watching, lunging, meowing). Officers were also critically important informants, as they have knowledge about animals including their biological health (by recording symptoms of illness or injuries such as wounds, abnormal skin condition, coughing) and behaviours (by observing and recording behavioural signs of fear such as hiding and barking and also positive behavioural signs such as playing). Our observations of animals and our conversations with officers about their knowledge of animals enhanced our understanding of how tensions arise for the animals who are the focus of officers’ work.

The aim of IE is to map and track people’s work to discover how institutionalised ideas and processes materialise in everyday practices. This approach can identify ways to revise organisational protocols and, where possible, amend laws to better serve the interests of the subjects of institutional practices. In this paper, those subjects are the animals who live in precarious circumstances, and also the operators and officers who are tasked with investigating the health and welfare of animals.

### Research participants

This research is part of a larger project for which the BC SPCA was the central research partner. The BC SPCA is authorised by the BC government to enforce the PCA Act (provincial animal protection law) although police and Royal Canadian Mounted Police can also enforce animal protection laws. The PCA Act also allows the BC SPCA to enter into contracts with municipalities to enforce local by-laws. Officers respond to calls of concern about animals across the province received by operators at a Provincial Call Centre (BC SPCA 2022a). In 2021, the BC SPCA received 78,134 calls and conducted 9,077 investigations (BC SPCA 2022b).

BC SPCA staff (administrators, managers, officers, operators and frontline animal shelter staff), as well as the animals involved were research participants. The primary author (KEK) is a long-serving volunteer with the organisation. Before the study began, we met with frontline shelter staff, officers and some administrators to discuss the research and answer questions.

We used the ethnographic methods of participant and naturalistic observation, interviews and document analysis (Campbell & Gregor 2002; DeVault & McCoy 2006) for eight months in 2019; follow-up interviews were conducted as required via telephone or virtually (Zoom Video Communications Inc, San Jose, California 2021) in 2020. IE studies often focus on frontline staff because they connect clients to institutional discourses and texts. In this study, frontline staff (especially animal protection officers) similarly connected clients (animals) to institutional texts by fitting animals into existing institutional categories, processes and definitions (DeVault & McCoy 2006). For instance, officers categorised animals according to their property status, health and welfare, and recorded information using standardised forms and the digital shelter database. In this paper, although we include data from administrators, managers and shelter staff, we focus on the work of operators and officers when they receive calls from the public who are concerned about animals.

### Observations, interviews and document analysis

After operators and officers provided written consent to participate in the research, KEK observed what they did when operators received calls from members of the public and when officers investigated those calls. During observations at the Call Centre or during ride-alongs, KEK recorded written fieldnotes (during ride-alongs, fieldnotes were only recorded in the vehicle before and after visiting locations connected to investigations). KEK, without including details that could identify cases, observed how operators and officers recorded information and how they entered information into the digital database. When observations involved members of the public, verbal consent was obtained as follows. Officers explained why they were visiting and that they had a student researcher (KEK) with them to observe and learn about their work for research purposes. After members of the public provided verbal permission for the officer and KEK to approach or enter, observations began. Verbal consent is permitted when written consent may be interpreted as untrustworthy by participants (TCPS2 2022, article 3.12; pp 60–61). Further, such observations are considered to be ‘minimal risk’ by the Tri-Council Policy Statement on the Ethical Conduct for Research Involving Humans because they do not allow for participant identification in dissemination of results, are not covert, are not staged by the researcher and are non-intrusive (TCPS2 2022, article 10.3; pp 191–193).

KEK conducted interviews during and after observations with operators and officers, asking them to explain the different steps they were taking and how they used physical and digital texts in their work. Texts play a critical role in institutions, and therefore within IE research, because they are central features of how institutions organise what people do. Texts include forms, checklists, notification documents, computer screens, and images (Smith & Turner 2014; p 5), together with laws such as municipal and provincial animal protection laws. KEK also had regular access to the digital shelter database (excluding confidential information). During interviews, KEK often asked follow-up questions about officers’ work processes, the information they recorded, how they used texts in their work, how they entered information into the database, and how they co-ordinated their work with other staff, officers and others.

### Data analysis

During data collection, we noted situations that seemed to cause tension, for example, if the outcome of a situation was not in the interests of the animals or people involved. Events surrounding Henry and the dogs he lived with seemed to cause such tensions, and thus we use their story as an entry-point to explore how such tensions arose.

As described by McCoy (2006; p 117), IE analysis involves first understanding what individuals are doing and experiencing in their daily work, and then analysing how these activities and experiences are organised by institutional processes. In this way, the institution, not the research participants, is the analytical focus (McCoy 2006; pp 109–110). The aim is to investigate how experiences of these individuals are being organised by ‘generalised institutional processes’, which is not the same as the conventional form of generalisation which is to posit from the experiences of one group of individuals to a larger population (Smith 1987; p 187). To discover the institutional processes, we identified institutional texts and practices that operators and officers followed and discussed (e.g. municipal laws, entering information into the database, issuing orders) when performing work activities (McCoy 2006; pp 111–115). We also talked with them about how they used texts to track animals and co-ordinate their work with other staff.

**Figure 1.**
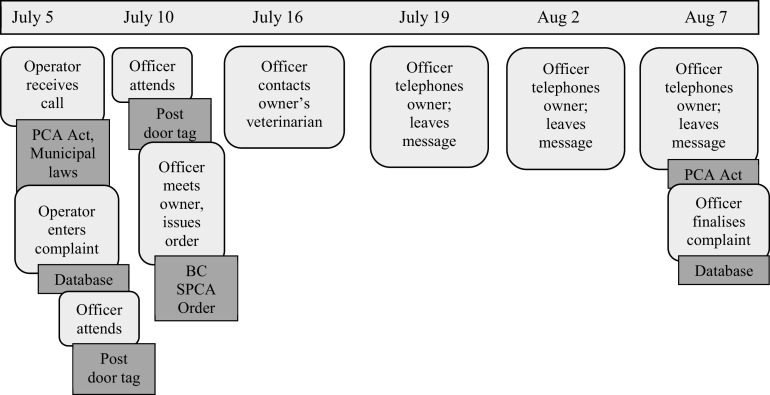
Chronological map detailing the texts and work processes activated by officers and Call Centre operators during Henry’s early life in 2014*. * Dark grey boxes with sharp edges are texts and light grey boxes with curved edges are work processes.

**Figure 2.**
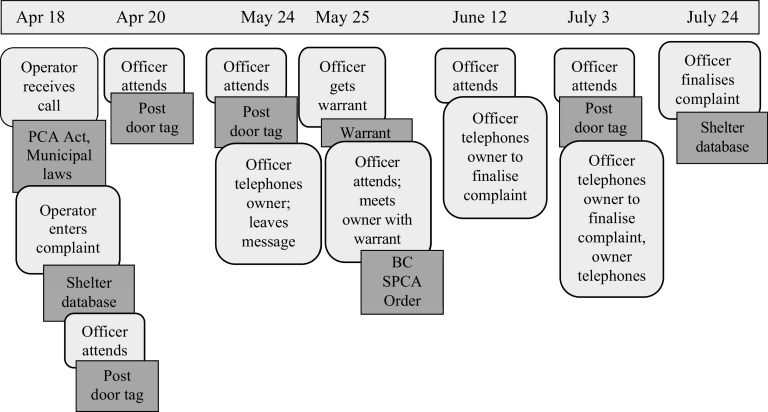
Chronological map detailing the texts and work processes activated by officers and Call Centre operators during Henry’s early life in 2015*. * Dark grey boxes with sharp edges are texts and light grey boxes with curved edges are work processes.

We followed IE analytical techniques described by Rankin (2017a,b) which include mapping and indexing. In order to orient ourselves to what happened in Henry’s early life when he and the other dogs were being investigated, we created a chronological map to show how and when work processes and texts were involved in investigations surrounding Henry. We then used observations and interviews from ride-alongs to ethnographically describe the work processes, and texts referenced on the map. To do this, we first ‘indexed’ (Rankin 2017a) the data. Indexing differs from qualitative coding techniques that develop themes via interpretations of what people do or say. Indexing categorises empirical descriptions of work processes. We listened to and transcribed audio-recorded interviews (in full or partially, depending on the relevance to tensions we were following) and inserted marginal comments in the transcribed document. Handwritten fieldnotes from observations were indexed using colour-coded tape. For instance, much of the work carried out by officers involved ‘attending’ and ‘posting’ as described in the *Results* and in Figures 1 and 2. This work included sub-indices of, for example, ‘reviewing and prioritising calls’ and ‘inspecting animal housing.’ We elaborated upon points on the map using indexed data from observations and interviews in order to stay grounded in how these processes occur and to provide examples.

Finally, to protect participant confidentiality in accounts and data, all names are pseudonyms, the pronoun ‘they’ is used, and we altered certain data (e.g. locations, dates, number of animals involved in cases) in a way that maintains the approximate features of events without compromising confidentiality. In the text, ‘I’ refers to KEK.

## Results

### Ethnographic account and maps

The ethnographic account and maps below describe a series of interactions between Henry and the BC SPCA in the early part of Henry’s life before he and the other dogs were seized and brought to a shelter. They provide a description of the dogs’ living environment and a broad introduction to the everyday work processes that operators and officers carry out when they receive calls from members of the public reporting concerns about animals.

*Upon review of Henry’s case, I found physical and digital documentation of different work processes and texts that had been used by operators and officers. A veterinary report completed during the seizure included photos of the property. Henry and the six other dogs had been living in squalor; floors and surfaces were thickly covered with layers of faeces, urine, mould, garbage and debris. One section of the floor was layered with empty dog food bags and while there was an empty bucket, food and water were not available. The report described the property, including high ammonia levels irritating the officers’ (and dogs’) lungs and eyes, overgrown bushes, and flies. Veterinary assessments reported that the dogs were mostly bright and alert but undernourished, had evidence of old scars on their faces and bodies, mites and suspected ringworm. The report noted that, based on the state of the property and dogs, the dogs were in ‘distress’ as defined by the PCA Act and thus officers removed the dogs from the property and brought them to a shelter.*

*Over the six years before the seizure, officers had visited Henry and the other dogs on multiple occasions. Members of the public telephoned the Provincial Call Centre and operators ‘entered complaints.’ After each call, officers ‘attended’ (visited the property) and ‘posted’ (left a door tag indicating the time of the visit) at the property. Attending and posting at properties were repeated multiple times for each call in an attempt to communicate via telephone and in person with the owner. When Henry was six months old, officers made contact and talked with the owner on their first visit to the property. When Henry was 1.5 years old, after three attempts to contact the owner, officers applied for and obtained a search warrant from a judge to search for and collect evidence of ‘distress’ at the property. For each complaint, after talking with the owner, officers ‘issued orders’ that detailed what the owner needed to do to alleviate the dogs’ ‘distress.’ Orders were ‘finalised’ a few weeks later in each case. Chronological maps (Figures 1 and 2) show the different work processes and texts activated by officers and operators during these time-periods.*

This account and accompanying maps chronicle the involvement of the BC SPCA and generate questions about the work processes and texts that organise and direct what operators and officers do in their daily work. The map raises questions regarding the multiple intervention attempts made by officers and the work of ‘finalising’ a case and what this means for animals. The account and maps thus provide an entry-point to explore a series of tensions that arise between the organisation of everyday operator and officer work and the interests of animals living in deprived situations. The tensions centre around the work processes of: (1) receiving and entering complaints; (2) looking for evidence of ‘distress’; and (3) responding to concerns not covered by the law.

### Receiving and entering complaints

We begin by explicating the work processes and texts that appear on the maps as: ‘operator receives call’ and ‘operator enters complaint.’

A large part of the work carried out by operators at the Provincial Call Centre involves receiving calls from members of the public who are concerned about an animal(s). I observed Lee, an operator, to see how this work is accomplished. Lee greeted each caller kindly and asked them to explain their concerns for the animal. One caller had concerns about a dog tethered outside that was left alone most of the time. Lee typed notes in a text document as they listened and then explained that most municipal animal control laws allow people to tether their dogs, but some include specifications. Lee needed more information and thus proceeded to ask the caller specific questions, listening and typing notes after each question:

*“Can you describe the yard?”*

*“What is provided for the dog? Food, water?”*

*“Can you describe the tether – what is it made out of? Chain, rope? Is it around the dog’s neck?”*

*“What about the area around the dog, are there faeces or other potentially harmful objects?”*

The caller confirmed that the dog had food, water, shelter, and the area was relatively clean. Lee then opened a computer folder, organised by municipality, with information about the local SPCA branches, veterinary clinics, and other services. Lee found the municipal law regarding dog tethering for the caller’s city and confirmed that what the caller described is permissible. The caller was still concerned, however, that the dog was left alone for most of the day, to which Lee responded:

*“I understand, but the law does not require people to spend a lot of time with or play with their animals. The law requires that the animal’s basic needs are met, food, shelter, water, things like that. Another concern could be matting – can you see how the dog’s coat looks?”*

Lee listened and after confirming with the caller that the coat looked ‘rough’, decided to enter the caller’s concerns as a complaint in the database. Thus, it was the report of the coat looking ‘rough’ and speculation that it could be matted, not the caller’s original concerns about the dog being left alone, that ultimately led to Lee entering the complaint.

Lee recorded the complaint officially in the database by entering information into different fields, for example, the dog’s physical characteristics and information about the animal’s presumed guardian. Lee copied notes from the text document into the file. Lee explained how they knew what to include:

*“We get a lot of calls about dogs left alone outside. When I trained with other operators, I learned what to ask, which words to use. A lot of it is just listening to people. They share more information that isn’t needed for the file. You have to narrow it down and just pick the details you need, like what the distress is, the specifics of the injury. Also dates and times are really important.”*

In summary, receiving and entering complaints is organised around determining whether an animal may be in ‘distress’ according to the legal definition in the PCA Act, whether municipal laws are being broken, and using specific criteria to determine whether ‘grounds’ exist for an officer to investigate. Through training with other operators and experiences, Lee and other operators know to listen to people and ask callers about these details. Specific criteria for what officially constitutes ‘distress’ are included on the official ‘BC SPCA Order’, which includes a list of actions that an individual must do to relieve ‘distress’ (e.g. providing water and shelter, clean area, coat care). Thus, the legal term ‘distress’ enters into their daily work and creates a tension between ‘distress’ as understood by the public (e.g. a social animal spending the majority of time alone) and the legal definition that needs to be met to justify intervention.

### Looking for and finding evidence of ‘distress’

In Henry’s case, when calls were received from members of the public, operators determined that there may be ‘distress’ and thus entered a complaint in the database (Figures 1 and 2). But the question remains, how did it happen that Henry and the other dogs repeatedly entered animal protection work processes over the course of two years when Henry was a puppy, but were not removed until he was older? Examining the work processes and texts that appear on the maps as: ‘officer attends’, ‘officer posts’ and ‘officer issues order’ provides an institutional analysis of how these events unfolded.

Officers investigate complaints (or ‘calls’) as a large part of their everyday work. On a ride-along, Officer Casey explained that at the beginning of their shift they review the database and prioritise emergency calls and calls that are causing more ‘distress’ to an animal. To support this triage work, operators sometimes telephone officers directly when they receive an emergency call. Casey reviewed the list, selected several calls to investigate, and we then departed the office to ‘attend’ the first call.

The call was about a dog confined in a doghouse in the backyard of a home. The caller reported that the dog was unable to leave the doghouse, and the caller could not see if the dog had access to water. When we arrived at the property, we knocked on the door but no one answered. *“Let’s wait a few minutes”* Casey said. While we waited, we walked through the alley to the back of the house to look for the dog, the doghouse, dishes, or toys. As we looked from the alley, Casey explained: *“We have to be careful and respect people’s property. And we have to get consent to go on their property.”*

As we looked, we identified the doghouse but no dog. We waited another five minutes and then Casey decided to ‘post’ on the property so we could continue to the next call. To ‘post’, Casey wrote their name, telephone number, time and ‘please call’ on a BC SPCA door tag and hung it on the front door. Back in the truck, Casey wrote a brief description of what we saw and did in their notebook. *“Hopefully they’ll call us soon”* Casey said while starting the truck to head to the next call.

This account of ‘attending’ and ‘posting’ is something officers carry out routinely. The actions Casey takes are guided by their knowledge of the need to respect property and privacy under the Canadian Charter of Rights and Freedoms as well as sections 13 and 14 of the PCA Act (sections which detail whether and when officers have authority to enter premises with and without a search warrant). While officers have the ‘right of inquiry’ under Common Law (i.e. the legal system used in commonwealth countries such as Canada), they must also follow the Charter which protects Canadians’ rights including that “everyone has the right to be secure against unreasonable search or seizure.” These legal requirements constrain what officers can do when investigating calls; they cannot go on the property to look thoroughly for the dog, and they have to give people time to respond to posts. If the person does not respond to the post, Casey will repeat the process of attending and posting in a few days, depending on the urgency of the situation (e.g. animal injury, weather conditions). Indeed, officers working on Henry’s case attended and posted multiple times, giving the owner time to respond to posts before talking to the owner, during which time the animals’ situation could not be assessed or addressed (Figures 1 and 2).

On another day, I accompanied Officer Bryce on a call about a dog on a long tether in the front yard of a home in a semi-rural area. The dog barked and ran at other dogs, children, people and cars when they passed. Upon arrival at the property, we saw the dog running back and forth barking at us. We knocked on the door, Bryce introduced us to the owner, explained the call and asked if we could see the dog. The owner agreed, and we approached the dog and saw a wooden shelter, food bowl, and a water bowl that lay overturned. The dog stopped barking and looked at us, panting heavily. Bryce asked the owner about their feeding and walking routine, explained the importance of developing a bond with the dog and suggested that a harness instead of a collar might be more comfortable for the dog.

Inspecting the area, Bryce noted that the shelter had no bedding and that the dog had no access to water. The owner responded that they would dig a hole for the water bowl so it would not tip over and also agreed to add straw on the shelter floor. Bryce then ‘issued an order’ which gave the owner an established mandate to complete these tasks. The mandated actions were established by Bryce checking the boxes on the order next to: “provide access to clean potable drinking water at all times” and “provide shelter that ensures protection from heat, cold and dampness appropriate to the protective outer coat and condition of the animal.” Bryce gave the owner one week to complete the tasks and told them that they could text photographs to provide the evidence that the order had been followed.

On the way to the next call, Bryce explained: *“We have to be clear about what we want people to do and give them chances to alleviate distress.”* Giving people chances to alleviate ‘distress’ is required by the PCA Act, section 11:

“If an authorised agent (i.e. officer) is of the opinion that an animal is in distress and the person responsible for the animal (a) does not promptly take steps that will relieve its distress, or (b) cannot be found immediately and informed of the animal’s distress, the authorised agent may, in accordance with sections 13 and 14, take any action that the authorised agent considers necessary to relieve the animal’s distress”.

Hence, Bryce needs to document the ‘chances’ (the time to remediate) that have been given and that a process has been followed (e.g. posting, attending, issuing orders). Adhering to this process must be documented in order for the BC SPCA to intervene further should the owner fail to follow the order in which case the animal could potentially be removed. Owners can formally appeal animal removal, thus initiating court proceedings to have the animal returned. Evidence of the process taken before removal and the ‘chances’ that have been given contribute to the court decision as to whether or not to return the animal to its owner. In the case above, where Bryce had issued an order, a few hours later, Bryce received a photograph from the owner that showed a large bucket in the ground filled with water, and a thick layer of straw in the doghouse. *“This is great,”* Bryce said while looking at the photographs. Bryce texted the owner to let them know they will visit again in a few days to ‘finalise’ the call, which includes confirming completion of the tasks and then writing in the database that the owner had followed the directions and no further action was required.

Bryce’s interaction with this owner, like Lee’s questions to the caller, is organised around the legal term ‘distress.’ In this case, Bryce can tick boxes on the BC SPCA order form where the legal definition of ‘distress’ is embedded. Later, Bryce documented the case in the database, briefly noting that they ‘issued an order’ and would follow-up in the coming days. After the follow-up visit, if the process ‘worked’ to elicit the owner’s compliance, Bryce would change the complaint status to ‘finalised’ in the database and type ‘no further action required’, officially closing the file. However, the caller’s original concerns about the dog being tethered outside, as well as Bryce’s own concerns and knowledge (their advice to the owner to the owner to spend time with the dog and to use a harness), are different from the concerns captured in the official order to provide water and adequate shelter. This reveals a tension between what is actually happening (a social animal tethered alone and displaying anxious behaviours) and the official account of the situation (‘distress’ alleviated through the provision of adequate shelter and water).

Henry’s case was less clear-cut than the one above. In 2015, officers issued an order after obtaining a search warrant (a court order that authorises a search, see Schedule A of the Prevention of Cruelty to Animals Regulation under the PCA Act) as there had been no communication from the owner after officers ‘attended’ and ‘posted’ three times. Obtaining the warrant is a documentary process whereby officers amass evidence (e.g. from the original call in the database, handwritten notes) and present ‘grounds for belief’ of ‘an animal in distress’ and a clear chronological record of the dates and times of each attempt to contact the owner (i.e. ‘attending’ and ‘posting’). Officers must establish that the owner has been given ample direction and time to address the animals’ condition and that reasonable grounds exist that warrant further investigation. In 2014 and 2015, orders were finalised due to ‘insufficient evidence to proceed’ and because the dogs ‘appeared in adequate condition’ from afar. Thus, for Henry and the other dogs, it is not clear whether the owner completed the mandated tasks or whether officers had to evaluate whether they had sufficient evidence or grounds to proceed with the investigation.

In summary, the finalising of these orders shows that officers are constrained in their capacity to act on the animals’ behalf. Officers must invest time in ‘attending’ and ‘posting’; they must follow procedures to establish evidence of ‘distress’ in order to obtain a search warrant or seize animals. These hurdles privilege property and privacy rights over investigating concerns about animals. This also means that the situations of animals living in deprived circumstances (e.g. lack of freedom, lack of social contact) may not be addressed by the current legal processes based on the legal definition of ‘distress.’

### Responding to concerns that are not covered by the law

A final observation illustrates how officers have concerns for animals in certain situations that go beyond the legal definition of ‘distress.’ While I was preparing to accompany Officer Morgan on a ride-along, we met some other officers in the office who knew I was a researcher and gave verbal consent for me to use their conversation in my fieldnotes. An officer brought up a call about a large-breed puppy that was four months old. They explained that this was the fifth time they had received a complaint from a member of the public about the puppy. The puppy spent most of its time alone on an outdoor patio and the most recent call was from someone concerned about the puppy being alone. Officers had previously ‘attended’ and ‘posted’ and ‘issued’ and ‘finalised’ orders to ensure the provision of water and shelter for the animal.

In their informal discussion at the outset of their shift, the officers considered how to proceed and acknowledged that the situation did not clearly fall under the legal definition of ‘distress.’ The discussion revealed the knowledge they have about animal growth and development, and the risks for a young dog if early intervention cannot be made. One officer expressed concern that the puppy was at a critical point in time regarding the development of social skills to be able to live successfully in a community where there are people, children, animals, bicycles, cars, and noises. A lack of social skills could result in the animal developing fearful or anxious behaviours and it may be difficult for the animal to adjust to new environments and stimuli in the future.

With a new call about the puppy, the officers reconsidered how to act on its behalf. The officers, concerned about the dog and the potential for future problems, decided (based on their cumulative experience) to speak with the owner, discuss the dog’s social development and see if the owner might relinquish the dog to the shelter. This sort of ‘soft’ approach involves a critically important set of work knowledge and skills that the officers applied when responding to concerns not covered by the law.

In this and similar situations, officers are balancing various fields of knowledge to guide their decisions and actions. One field is organised around the legal definition of ‘distress.’ Another is grounded in their knowledge about dog behaviour and social development, their concern that this dog might not be developing important social skills, and their knowledge that this failure can have later consequences for the dog and people. Another field of knowledge is less obvious: the work knowledge they have about how to proceed in such cases. This includes knowledge about the context related to the owners’ prior responses, the veracity of the new complaint, and the interpersonal skills they possess that are critical to securing a voluntary ‘relinquishment.’ While the dog was not technically in ‘distress’, officers recognised that it could develop behavioural problems and considered what other actions they could take to help the animal. Thus, there is tension between these different types of knowledge because officers are concerned about the dog yet they understand that the actions they can take are constrained by property and privacy law.

## Discussion

The interests of animals such as Henry living in deprived situations, and the concerns that operators, officers and members of the public have for these animals, are at odds with authorised institutional practices guided especially by the legal definition of ‘distress.’ When Henry and the dogs he lived with were young, they were investigated by officers, but investigations did not lead to their removal from the environment until much later. Such delays can involve costs to animals in deprived situations as they can develop behavioural problems that are difficult to rehabilitate in a shelter. This can also involve costs to operators and officers as they face constraints in their work when determining when and how they can take action to protect animals and, in some cases, officers must be strategic and creative as they develop other ways to respond to concerns.

Our in-depth examination of the actual work of officers and operators, and of the tensions that arise in their work, differs from approaches used in the small amount of previous research on animal protection law enforcement. However, Arluke’s (2004) description of how dispatchers (i.e. operators) listen for ‘key words’ that signal cruelty and ask callers to provide details (e.g. whether the animal looks ill, whether food is available) is similar to IE’s focus on everyday work. Additionally, Arluke (2006) discussed officers’ efforts to educate people to be “more responsible animal owners.” However, Arluke’s efforts to categorise and conceptualise such activities (for example, by classifying officers as either ‘animal-inclined’ or ‘police-oriented’ based on their ‘style’ of enforcement; Arluke 2004) differs from IE where the task is to discover the social organisation of these practices rather than theorise about them. In another example, Irvine (2003) presented disjunctures between how animal sheltering institutions ‘think’ about the needs of human clients (e.g. desire to have a lifelong animal companion) versus their lived experiences (e.g. preference to relinquish an animal). This work is also ethnographic, however, it connects findings that suggest an abstracted form of agency to a concept termed institutional ‘thinking’ (i.e. organisational discourse and framing). In contrast, our study stayed focused on the actual actions and work knowledge of operators and officers themselves, including the questions they ask, their efforts to apply the law in the interests of animals and the ‘soft’ approaches they sometimes use.

Other studies such as Stull and Holcomb’s (2014) survey research with animal protection officers and Coulter and Fitzgerald’s (2019) combination of surveys and interviews suggest similar tensions to those described here. As well, Coulter and Campbell (2020) used interviews and document analysis to create a map of animal protection law enforcement in Manitoba, Canada. In another case, Holmberg (2014) interviewed animal welfare officers and analysed how they made judgements about hoarding situations using visual (e.g. photographs), olfactory (e.g. ammonia levels) and auditory (e.g. dogs barking) information.

Aligned with the concerns about animals described by this small group of researchers, we too examined tensions in the work of animal protection officers. Our research focused on how such tensions arise within the ‘organised’ practices (i.e. those dictated by legal documents and the organisation’s own procedures) that are embedded in the work. Our analysis is focused on understanding whether and how the work of entering complaints, issuing orders and finalising cases could be modified to better protect animals. Currently, the work of animal protection officers is constrained by the need identify some aspect of the situation that may meet the legal definition of ‘distress’, for example, when Lee entered a complaint about matted hair, and when Officer Bryce issued an order to provide straw and a well-anchored water bowl. However, these interventions did not address the original concerns about social isolation, lack of stimulation, and the well-being of an agitated dog kept outdoors on a tether.

These tensions arise from the legal definition of distress in the PCA Act which limits when officers can intervene on behalf of animals. ‘Distress’ is cited (but defined in different ways) in most provincial and territorial animal protection laws in Canada. The definition has also broadened over time in some provinces (Fraser *et al.* 2018). For example, in 1997 BC’s PCA Act considered an animal to be in distress if it was “deprived of adequate food, water, shelter, ventilation, space, care or veterinary treatment, injured, sick, in pain or suffering, or abused or neglected.” In 2012, this definition was expanded (and remains today) to include deprivation of light and exercise, being “kept in conditions that are unsanitary” and being “not protected from excessive heat or cold” (British Columbia Government 2023).

Despite this broadening of the definition of ‘distress’, the constraints created by the term prevented officers from dealing with certain concerns where members of the public expected the BC SPCA to intervene. Given that the term ‘distress’ guided and limited operator and officer work in BC, and that other jurisdictions define ‘distress’ somewhat differently, future research could observe enforcement work in different jurisdictions to understand how different definitions influence what officers can and cannot do. For example, in Nova Scotia’s Animal Protection Act the definition of distress now includes “suffering undue … anxiety” and “kept in conditions that are unsanitary or that will significantly impair the animal’s health or well-being over time.” These inclusions could conceivably allow officers to take early action in cases of social deprivation such as Henry’s deprived early environment.

Another tension arose for officers as they followed specific procedures that privileged property and privacy rights over concerns about animals. Figures 1 and 2 show the routine nature of attending and posting work as the PCA Act requires officers to give people time to alleviate distress. The result is that early intervention is difficult to achieve and that animals such as Henry can remain for years in a deprived environment without opportunities for normal development and socialisation even though such opportunities are widely recognised as being important (McCune 1995; Cutler *et al.* 2017; Association of Shelter Veterinarians 2022). Animals so deprived may ultimately end up in shelters, as eventually happened in Henry’s case. In such cases, shelter staff are challenged to modify the animal’s behaviour so that adoption is possible. The current social organisation of animal protection work results in officers who lack authority to intervene meaningfully before problems develop. Here, again, policies, procedures and legal authority might usefully be reviewed and revised, perhaps aiming to re-balance the interests of animals versus the property and privacy rights of owners.

The final tension we note here is the glimpse of the time-consuming ‘informal’ work activities that officers undertake to protect animals. These are activated in response to concerns not covered by the law. We presented the example of operators receiving similar calls about the same puppy. After issuing and finalising multiple orders, the officers informally consulted about how to proceed within the limitations of the law. Their formal knowledge about what legally constitutes ‘distress’ was integrated with their knowledge of the case (prior calls and animal development and behaviour) and their work knowledge of what might facilitate a solution. Together, these enabled them to initiate an ‘*ad hoc*’ plan to address the dog’s welfare.

## Animal welfare implications and conclusion

In the organisation we studied, when and how operators and officers can take action for animals is constrained by the need to identify concerns that fall within the legal definition of ‘distress’, and by the priority given to privacy and property rights in investigating concerns. As well, action was directed towards providing evidence of owners being given ‘chances’, should the case be taken to a court appeal. These socially organised constraints direct officers and operators to spend much of their time determining whether calls have ‘grounds’ for investigation, ‘posting’ and ‘attending’ at properties and also ‘issuing orders’ when ‘distress’ is identified. These constraints make it difficult for officers to intervene for many animals including those living in deprived situations. Avenues for further inquiry and possible improvement include:

- The definition of distress could be expanded to include conditions that are likely to lead to future harm. This could empower staff to intervene earlier when animals are at risk.
- Altering the balance between the interests of animals versus the property and privacy interests of owners could allow more prompt intervention.
- Similar IE work in jurisdictions differing in culture, geography and language could determine how different legislation and systems of enforcement organise what happens to animals.
